# EvoSubster: a pipeline for evolutionary inference of single- and double-base substitution spectra

**DOI:** 10.1093/bioadv/vbag154

**Published:** 2026-06-01

**Authors:** Mariko Nakagawa, Martin C Frith

**Affiliations:** Department of Computational Biology and Medical Sciences, The University of Tokyo, Kashiwa, Chiba 277-8568, Japan; Department of Computational Biology and Medical Sciences, The University of Tokyo, Kashiwa, Chiba 277-8568, Japan; Artificial Intelligence Research Center, National Institute of Advanced Industrial Science and Technology (AIST), Koto-ku, Tokyo 135-0064, Japan

## Abstract

**Motivation:**

Mutational processes differ widely across the tree of life, yet most existing resources focus on somatic mutations in humans or on a limited set of well-studied species.

**Results:**

We present EvoSubster, a simple and extensible pipeline for inferring evolutionary single-base and double-base substitution spectra from closely related species using a parsimony-based three-genome comparison. The pipeline automatically downloads NCBI genomes, aligns them, infers substitution direction, quantifies single-base and double-base substitutions, and outputs visualizations. Applying EvoSubster to diverse fungal and cnidarian genomes revealed distinct lineage-specific substitutional signatures, including TTA>TCA and TTA>TGA in mushroom-forming fungi within Agaricomycetes, ACA>AAA and ACG>AAG in cnidarians, CG>TT and GC>AA in Mucoromycota, and frequent A: T-rich adjacent substitutions in Glomeromycetes (arbuscular mycorrhizal fungi).

**Availability and Implementation:**

EvoSubster is implemented as a set of Python 3, R, and bash scripts and is freely available on GitHub at: https://github.com/marikie/EvoSubster. The pipeline relies on a small number of easy-to-install, publicly available command-line tools. Installation instructions and example workflows are provided in the online documentation.

## 1 Introduction

Nucleotide substitution patterns provide essential insights into genome evolution, mutational mechanisms, and DNA repair processes, yet most existing work has focused on somatic mutations in human cancers (Koh et al. 2021) and germline mutations in a limited number of intensively studied organisms (Bergeron et al. 2023, Ruis et al. 2023), leaving substitution spectra across the broader diversity of life poorly characterized in an evolutionary context. Comparative analyses of closely related genomes offer a powerful framework to detect context-dependent substitutional tendencies. Here, we present EvoSubster, a generalizable, automated, and reproducible pipeline that applies a parsimony-based three-genome comparison to infer evolutionary single-base and double-base substitution spectra, enabling researchers to analyse substitution spectra in their taxa of interest.

## 2 Methods

Our pipeline’s input should be NCBI genome accession IDs of three closely related species of your choice (we suggest >80% identity of orthologous DNA): species A as an outgroup, species B, and species C (Supplementary Methods, section “Selection of three close species”). It downloads the corresponding genomic FASTA files and, when available, gene annotations. Pairwise alignments between A–B and A–C are computed with LAST (Frith and Kawaguchi, 2015) and merged into three-way alignments. We restrict the analysis to ungapped columns and count substitutions in a strand-independent manner (e.g. A > G ≡ T > C). For quality control, EvoSubster also reports pairwise sequence identity between the input genomes, allowing users to verify that their chosen species are sufficiently similar for reliable parsimony-based inference.

**Figure 1:**
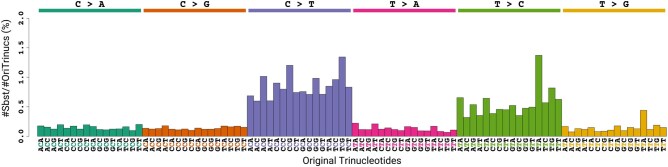
Single-base substitution spectrum inferred by EvoSubster for *Lentinula novae-zelandiae* (New Zealand shiitake mushroom). Bars show the frequency of each single-base substitution normalized by the number of occurrences of the corresponding ancestral trinucleotide. The spectrum shows elevated C > T and T > C substitutions, with particularly high TTA>TCA and TTA>TGA rates relative to other trinucleotide contexts.

Substitution direction is inferred by parsimony in each alignment column (Supplementary Methods, section “Single-base substitution analysis” and “Double-base substitution analysis”). Briefly, if only B differs from the shared base of A and C, we count a substitution in the B lineage; conversely, if only C differs from A and B, we count a substitution in C. Columns where all three species differ, or where the outgroup shares no clear ancestral state with either ingroup, are discarded as ambiguous. For each lineage, we aggregate the inferred events into context-dependent spectra. The single-base substitutions are visualized on a linear scale (Fig. 1), and also on a log scale that better shows low frequencies (Fig. S1, available as supplementary material at Bioinformatics Advances online).

### 2.1 Single-base substitution analysis

For single-base substitutions, we consider trinucleotide contexts in which the two flanking bases are conserved while the central base changes. For each ancestral trinucleotide, substitution rates are calculated as the number of inferred substitutions divided by the number of occurrences of that trinucleotide in the ancestral state (as inferred from the alignment).

### 2.2 Double-base substitution analysis

For double-base substitutions, we apply the same framework to four-base windows, requiring that the outer bases are conserved while both internal bases change. We summarize these events as double-base substitution spectra and normalize by the background frequency of the ancestral dinucleotides.

## 3 Results

We applied EvoSubster to a curated set of fungal and cnidarian genomes. We focused primarily on non-coding regions to mitigate the influence of strong functional constraints on substitution patterns, allowing us to better capture intrinsic mutational biases (Supplementary Methods, section “Use of non-coding regions for substitution inference”). Despite performing the full pipeline from genome download to spectrum visualization, wall-clock running times were modest, ranging from about 3 to 45 min per trio (Table S7, available as supplementary material at *Bioinformatics Advances* online).

### 3.1 Overall substitution trends

Across trios, we frequently observed high frequencies of C > T (or G > A) and T > C (or A > G) substitutions, consistent with the well-known transition bias favoring mutations between purines and between pyrimidines (Wakeley 1996). This trend was apparent in many fungal and cnidarian species in our dataset.

We also frequently observed high frequencies of CG>TG substitutions. This is consistent with previous reports that cytosines in CpG dinucleotides are hypermutable and frequently give rise to CG>TG substitutions (Cooper et al. 2011).

### 3.2 Lineage-specific single-base signatures

Beyond these general patterns, EvoSubster revealed several distinct lineage-specific single-base substitution signatures across fungal and cnidarian genomes.

#### 3.2.1 TTA>TCA and TTA>TGA in mushroom-forming fungi

A unique trend was observed in some mushroom-forming fungi within Agaricomycetes (e.g. *Lentinula novae-zelandiae*, *Laccaria trichodermophora*, *Agaricus bitorquis*): the frequencies of TTA>TCA and TTA>TGA substitutions were strikingly high (Fig. 1). In our dataset, this pattern was statistically significant and was observed specifically in Agaricomycetes: chi-square tests of trinucleotide context dependence produced $p<10^{-10}$ in all cases, with TTA>TCA and TTA>TGA ranking first in standardized residuals in 27 of 36 and 29 of 36 Agaricomycetes ingroup species, respectively, while none of the 18 non-Agaricomycetes ingroups showed this enrichment (Table S1, available as supplementary material at *Bioinformatics Advances* online; Supplementary Methods, section “Statistical Test”).

Extensive research on human skin cancers has revealed that UV-induced mutations typically show a strong C > T signature at dipyrimidine sites, but recent work has highlighted TTA>TCA as a prominent substitution in yeast under UVA exposure (Laughery et al. 2024). In humans, T > C mutations are rarely observed because DNA polymerase η (Pol η/XPV) accurately copies across UV-induced DNA lesions (such as TT dimers) without introducing mutations. By contrast, the corresponding enzyme in yeast, Rad30, is more error-prone and generates frequent T > C mutations—particularly TTA>TCA—within TT sequence contexts. This raises the possibility that these Agaricomycetes may possess a Pol η-like enzyme with Rad30-like error properties, contributing to the elevated TTA>TCA rates.

Although T > C mutations are generally rare among somatic mutations in human skin cancers, population genomic data show that TTA>TCA is among the more frequent TTA-derived substitutions in humans, with TTA>TGA also detected but at lower frequency (Prendergast et al. 2019). It is unclear whether these human patterns are mechanistically related to the striking TTA>TCA and TTA>TGA enrichments we observe in Agaricomycetes, and TTA>TGA in particular has been rarely reported and remains poorly understood.

#### 3.2.2 ACA>AAA and ACG>AAG in cnidarians

We also observed a general trend in several cnidarian species (e.g. *Acropora hyacinthus*, *Cassiopea xamachana*, *Montipora efflorescens*) in which ACA>AAA and ACG>AAG substitutions were elevated (Fig. S1, available as supplementary material at *Bioinformatics Advances* online). These patterns are statistically robust: chi-square tests of trinucleotide context dependence yielded $p<2.2\times 10^{-308}$ for every cnidarian ingroup examined, and ACA>AAA and ACG>AAG ranked as the top two contexts by standardized Pearson residual within the C > A category in 31 of the 32 ingroups across 16 trios (Table S2, available as supplementary material at *Bioinformatics Advances* online; Supplementary Methods, section “Statistical Test”).

### 3.3 Lineage-specific double-base substitution signatures

#### 3.3.1 CG>TT in *Podila humilis*, and GC>AA in *Podila verticillata*

In the soil fungal species *Podila humilis*, the most frequent double-base substitution from “CG” was “CG>TT,” in which the first base change (C > T or G > A) represents a transition, whereas the second base change (G > T or C > A) represents a transversion (Fig. S2, available as supplementary material at *Bioinformatics Advances* online). Statistical tests showed that substitutions from CG were significantly non-uniform in both the non-coding and whole-genome analyses ($p<2.5\times 10^{-6}$ and $p<1.9\times 10^{-6}$, respectively), with CG>TT showing the strongest enrichment (Table S3, available as supplementary material at *Bioinformatics Advances* online; Supplementary Methods, section “Statistical Test”). A similar tendency was observed in *P. verticillata*; however, in this species the bias was significant only in the whole-genome analysis ($p=3.99\times 10^{-3}$) (Table S3, available as supplementary material at *Bioinformatics Advances* online; Supplementary Methods, section “Statistical Test”).

Similar CG>TT substitutions have been reported at CpG sites in mammalian genomes, where cytosine oxidation or misrepair at methylated CpG can lead to tandem substitutions (Lee et al. 2002). Although fungal DNA methylation systems are diverse and remain incompletely characterized, particularly in under-sampled lineages such as Mucoromycota (Nai et al. 2021), the CG>TT enrichment observed in Podila raises the possibility that methylated CpG-associated damage or repair may contribute to this pattern.

Interestingly, we did not observe markedly elevated CG>TG single-base substitutions in these species, even though CpG sites are generally prone to C > T transitions. This may indicate that, in these lineages, CpG lesions are processed in ways that favor double-base changes, such as CG>TT, rather than being resolved as simple C > T transitions.

In *P. verticillata*, GC>AA was the most frequent double-base substitution from “GC” (Fig. S2, available as supplementary material at *Bioinformatics Advances* online). This bias was strongly supported in both the non-coding and whole-genome analyses ($p<5.7\times 10^{-7}$ and $p<5.8\times 10^{-10}$, respectively), with GC>AA showing the largest positive standardized residual (Table S3, available as supplementary material at *Bioinformatics Advances* online; Supplementary Methods, section “Statistical Test”). Error-prone DNA polymerase ζ, acting during translesion synthesis, has been reported to generate adjacent-base substitutions such as GC>AA (Seplyarskiy et al. 2015). The elevated GC>AA rate in this species might therefore reflect a polymerase ζ-mediated process.

#### 3.3.2 A: T-rich adjacent substitutions in arbuscular mycorrhizal fungi

**Figure 2:**
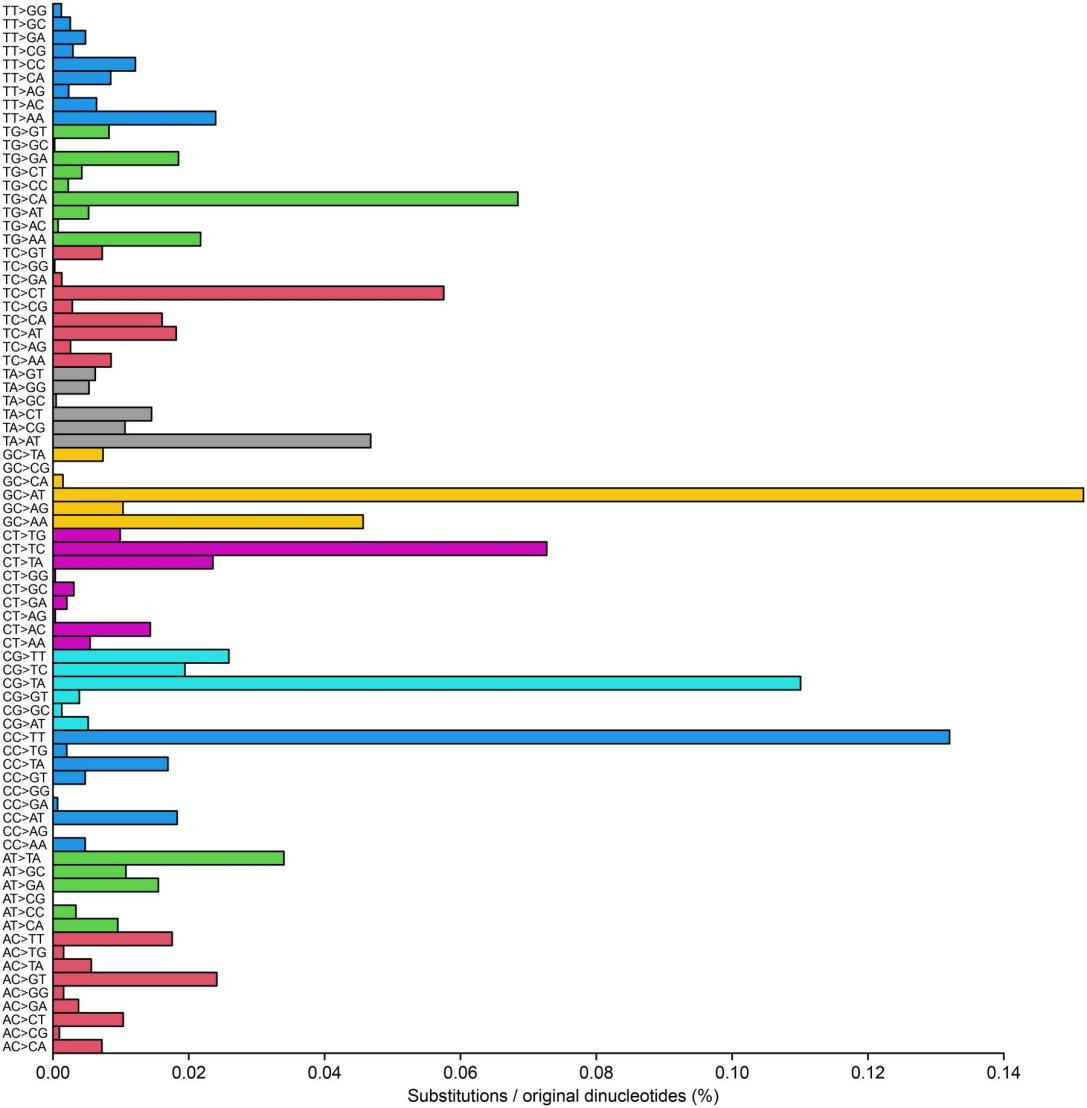
Double-base substitution spectrum inferred by EvoSubster for the fungus *Funneliformis caledonium*. Among A: T-rich dinucleotide contexts (TT, TA, and AT), the most frequent substitutions are the double transversions TT>AA, TA>AT, and AT>TA, indicating an unusual enrichment of A: T-to-A: T double transversions rather than transition-dominated patterns.

Glomeromycetes (arbuscular mycorrhizal fungi) such as *Gigaspora rosea*, *Funneliformis caledonium*, and *Rhizophagus irregularis*, showed distinct A: T-rich adjacent substitution patterns rather than double-base transition patterns (Fig. 2). These A: T-rich adjacent substitution patterns were also statistically robust. Across multiple Glomeromycetes species, chi-square tests of double-base substitution bias gave highly significant *P*value (all $p<10^{-10}$), and TT>AA, TA>AT, and AT>TA repeatedly showed the strongest positive standardized residuals within the TT, TA, and AT categories, respectively (Supplementary Tables S4, available as supplementary material at *Bioinformatics Advances* online; Supplementary Methods, section “Statistical Test”).

## 4 Conclusion

Taken together, these examples illustrate how EvoSubster can recover both well-known substitutional tendencies (such as CpG-associated C > T transitions) and more unusual lineage-specific signatures, including TTA>TCA/TTA>TGA in Agaricomycetes, ACA>AAA/ACG>AAG in cnidarians, and distinctive double-base patterns in Mucoromycota and Glomeromycetes. Because the pipeline is general and relies only on genome assemblies for closely related species, it can be readily applied to many other taxa to explore the diversity of evolutionary substitution spectra.

## Supplementary Material

vbag154_Supplementary_Data

## Data Availability

No new data were generated or analysed in support of this research.
