# Supplementary material for: EvoSubster: a pipeline for evolutionary inference of single- and double-base substitution spectra
Source: Bioinform Adv. 2026 Jun 1;6(1):vbag154. doi: 10.1093/bioadv/vbag154 (PMC13387360; doi:10.1093/bioadv/vbag154)
Supplement: vbag154_Supplementary_Data [file vbag154_supplementary_data.pdf]

Supplementary Methods

We investigated trinucleotides to characterize single-base substitution patterns and tetranucleotides to characterize double-base substitution patterns. We used DNA sequences of three closely related species (species A, B, and C), with species A as the outgroup, obtained from NCBI. We first aligned species A with B and A with C using LAST (28), and then merged these pairwise alignments into a multiple alignment. We scanned every trinucleotide in the whole-genome alignment for the single-base substitution analysis, and every tetranucleotide for the double-base substitution analysis. Substitutions in species B and C were inferred by parsimony (29). The analysis was restricted to ungapped columns, and substitutions were counted in a strand-independent manner (e.g. A>G ≡ T>C). We followed the convention used in cancer mutational signature studies for the choice of strand on which to represent substitution patterns (3,30). Substitution rates were calculated as the number of inferred substitutions divided by the number of original trinucleotides (or dinucleotides), using this denominator to reflect the background frequency of each context.

Single-base substitution analysis

Single-base substitutions were defined as events in which only the central base of a trinucleotide changes. Accordingly, we considered trinucleotide contexts in which the two flanking bases are conserved while the middle base varies. We classified each trinucleotide site into five cases: Case 0, all middle bases are identical in A, B, and C; Case 1, only A differs; Case 2, only B differs; Case 3, only C differs; and Case 4, all three middle bases differ (Figure S0).

In Case 0, we incremented the count of the original trinucleotide for both species B and C, as no substitution was inferred. In Case 1, two equally parsimonious scenarios are possible. Suppose the middle base in species A is ▲ and the middle base in both species B and C is ▽. One possibility is that the common ancestor possessed ▲, which later changed to ▽ in the ancestor of species B and C. Alternatively, the common ancestor may have had ▽, and the change to ▲ occurred in species A. Since it is not possible to distinguish between these two scenarios with confidence, this case was considered ambiguous and excluded from further analysis. In Case 2, the most parsimonious explanation is that the common ancestor had ▽, and a substitution to ▲ occurred only in species B. In this case, we incremented the count of the original trinucleotide for both species B and C, and incremented the substitution count for the change ◯▽□ → ◯▲□ in species B. Case 3 was handled analogously, with the substitution occurring only in species C. Case 4 was excluded due to its complexity; the presence of three different middle bases results in multiple equally plausible evolutionary scenarios, making it difficult to confidently infer the direction or occurrence of substitutions.

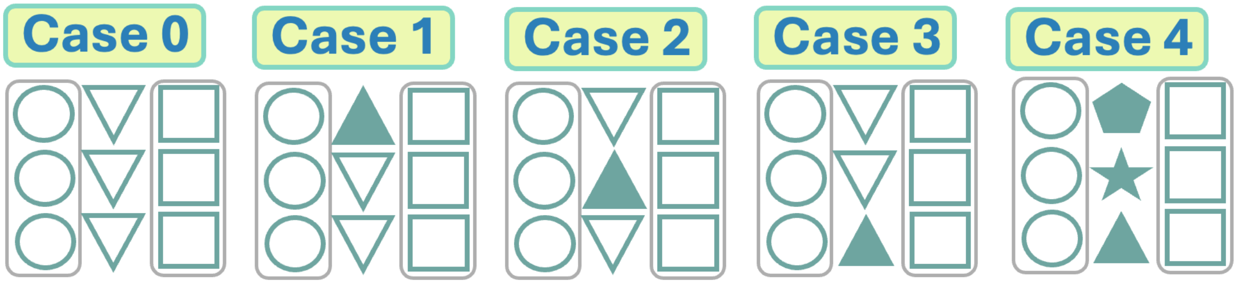

Figure S0. Five cases of trinucleotide contexts.

Double-base substitution analysis

To characterize double-base substitutions, we scanned the genome using four-base windows in which the first and fourth positions are identical across species, while both internal bases differ. These events were then aggregated

into dinucleotide substitution spectra and scaled by the background frequencies of the corresponding ancestral dinucleotides.

Selection of three close species

To apply a parsimony-based framework, we selected triplets of closely related species in which pairwise sequence divergence is low. The outgroup was chosen to be close enough for parsimony to remain reliable, but sufficiently diverged from the other two species that the species-tree topology ((B, C), A) is unambiguous. This is important because mis-specifying the outgroup can substantially change the inferred number of substitutions assigned to each lineage.

To formalize this, consider a species tree with three species: A, B, and C, where A is the outgroup, and B and C form the ingroup.

Let Id_{AB}, Id_{AC}, and Id_{BC} denote the pairwise sequence identities between species A and B, A and C, and B and C, respectively. Here, sequence identity is defined as the proportion of aligned sites at which the two sequences share the same nucleotide, e.g.

$$\mathrm{Id}_{AB} = \frac{\#\{\text{aligned sites where A and B have the same base}\}}{\#\{\text{aligned sites between A and B}\}}$$

The corresponding mismatch rates are then

- between A and B: $x = 1 - \mathrm{Id}_{AB}$,
- between A and C: $y = 1 - \mathrm{Id}_{AC}$,
- between B and C: $z = 1 - \mathrm{Id}_{BC}$.

Let p_A, p_B, and p_C denote the probabilities of mutation along the branches from the tree's internal node to A, B, and C, respectively. Under a simple additive model of divergence, we can write

- $x = p_A + p_B$
- $y = p_A + p_C$
- $z = p_B + p_C$

which gives

- $p_A = \frac{x+y-z}{2}$
- $p_B = \frac{x+z-y}{2}$
- $p_C = \frac{y+z-x}{2}$.

For each ingroup species (B and C), we then compared the probability that a site pattern is explained by a single substitution (parsimony-consistent) versus two substitutions (non-parsimonious), under the assumption that any of the three non-ancestral nucleotides is equally likely (factor 1/3).

Species B (ingroup)

- Probability of a parsimony-consistent scenario (one substitution on branch B): $p_{\text{pars}}(B) = (1 - p_A)\,\frac{p_B}{3}\,(1 - p_C)$

- Probability of a non-parsimonious scenario (two substitutions on branches A and C): $p_{\text{nonpars}}(B) = \frac{p_A}{3}\,(1 - p_B)\,\frac{p_C}{3}$

Thus, the ratio of parsimony-consistent to non-parsimonious scenarios for species B is

$$\text{ratio}_\text{B} = 3 \cdot \frac{(1 - p_A)p_B(1 - p_C)}{p_A(1 - p_B)p_C}$$

Species C (ingroup)

- Probability of a parsimony-consistent scenario (one substitution on branch C): $p_{\text{pars}}(C) = (1 - p_A)\,(1 - p_B)\,\frac{p_C}{3}$
- Probability of a non-parsimonious scenario (two substitutions on branches A and B): $p_{\text{nonpars}}(C) = \frac{p_A}{3}\,\frac{p_B}{3}\,(1 - p_C)$

The corresponding ratio for species C is

$$\text{ratio}_\text{C} = 3 \cdot \frac{(1 - p_A)(1 - p_B)p_C}{p_A p_B(1 - p_C)}$$

These calculations were used as a rough check to confirm that, for the chosen species triplets, parsimony-consistent histories for the ingroup branches (B and C) are expected to be much more frequent than non-parsimonious two-hit histories.

In practice, we selected three species whose pairwise sequence identities were all above 80%. To ensure a reliable outgroup assignment, trio exclusion is based on a two-step rule: if the genus pattern matches the target configuration (species A differs from both B and C while B and C share a genus), the trio is always retained, assuming that the original taxonomic classification reflects greater divergence. Otherwise, we examine substitution identities: when $\text{Id}_{\text{AB}} < \text{Id}_{\text{BC}}$ and $\text{Id}_{\text{AC}} < \text{Id}_{\text{BC}}$ hold, the trio is retained unless the genera form a two-vs-one configuration (species A shares a genus with only one ingroup species), in which case it is excluded.

Use of non-coding regions for substitution inference

To reduce the confounding effects of purifying selection on coding sequences, substitutions were inferred primarily from non-coding regions. When genome annotation files for species A were available, coding exons were excluded based on those annotations. For each trio, only the annotation file for species A was used. This approach was adopted for two reasons: first, because the species are closely related, most regions are expected to retain the same coding or non-coding status across the three genomes; second, because genome annotations are imperfect, differences in annotation quality among species could introduce additional bias if annotations from species B and species C were also used.

Statistical Test

To test whether substitution rates depend on local sequence context, we analyzed single-base and dinucleotide substitutions using context-specific tests of homogeneity. For single-base substitutions, each of the six pyrimidine-centered mutation classes (C>A, C>G, C>T, T>A, T>C, and T>G) was tested independently across trinucleotide contexts. For dinucleotide substitutions, each of the ten ancestral dinucleotide classes was tested independently.

For each focal substitution class, we constructed a 2 × k contingency table. The first row contained the number of sites at which the focal substitution was observed, and the second row contained the number of sites with the same ancestral central base (for single-base substitutions) or ancestral central dinucleotide (for dinucleotide substitutions) at which the focal substitution was not observed, i.e. sites where the central base(s) either remained unchanged or changed to a different alternative state. The k columns represented all the substitution patterns (we excluded patterns with zero total counts for the test). Thus, the null hypothesis was that the probability of the focal substitution was constant across all substitution patterns, and that any apparent variation in substitution rate among contexts was due to chance alone.

When all expected cell counts were at least 5, we applied a Pearson chi-square test of homogeneity ($df = k - 1$). When any expected cell count was below 5, we instead used Fisher's exact test with a Monte Carlo approximation of the p-value based on 10,000 simulated tables. Significance was evaluated at $p = 0.05$ and $p = 0.01$. To identify which contexts were enriched or depleted for the focal substitution, we calculated standardized Pearson residuals for the substituted-site row. Positive residuals indicate contexts with more substitutions than expected under the context-independent null model, whereas negative residuals indicate contexts with fewer substitutions than expected.

Supplementary Fig. S1.

Log-scale single-base substitution spectra for cnidarian species: *Acropora hyacinthus*, *Cassiopea xamachana*, and *Montipora efflorescens*. Each point represents a trinucleotide substitution type (e.g. ACA>AAA) and shows the $\log_2$ of its substitution rate relative to the overall mean substitution rate across all substitution types (y-axis: $\log_2$[(# of substitutions / # of original trinucleotides) ÷ mean(# of substitutions / # of original trinucleotides) across all substitution types]). Positive values indicate substitution types with enriched rates, whereas negative values indicate depleted types.

Overall, the spectra are dominated by C>T and T>C substitutions, as commonly observed across many species, but the C>A class also shows noticeably elevated ACA>AAA and ACG>AAG substitutions, which is less typical.

For *Cassiopea xamachana* and *Montipora efflorescens*, gene annotations were not available, so the spectra include both coding and non-coding regions. In other annotated species, spectra from all regions and from non-coding regions alone were very similar, so we expect comparable patterns in these cnidarians as well.

Acropora hyacinthus

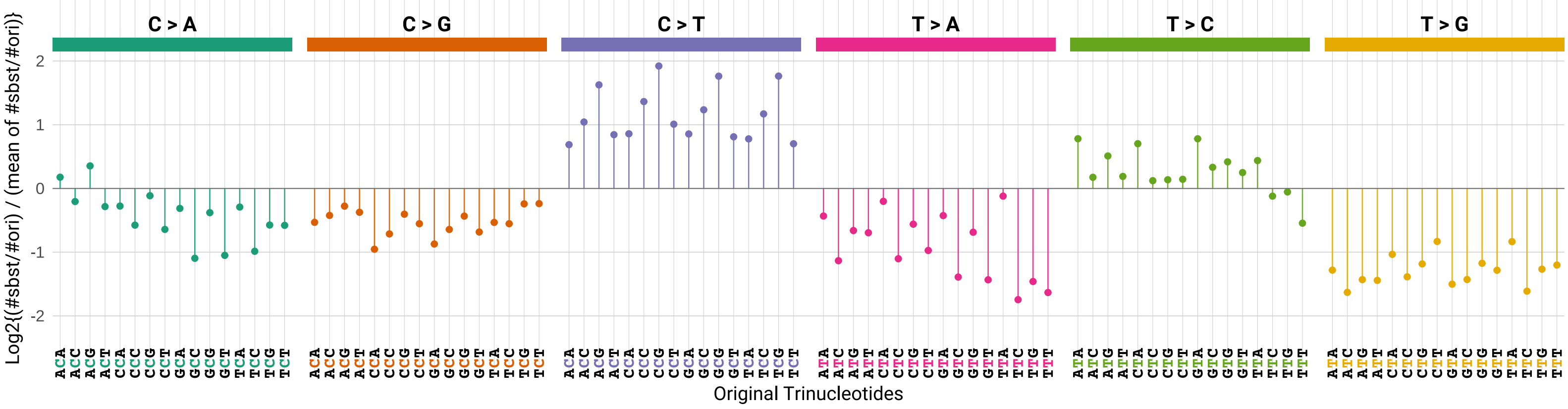

Cassiopea xamachana

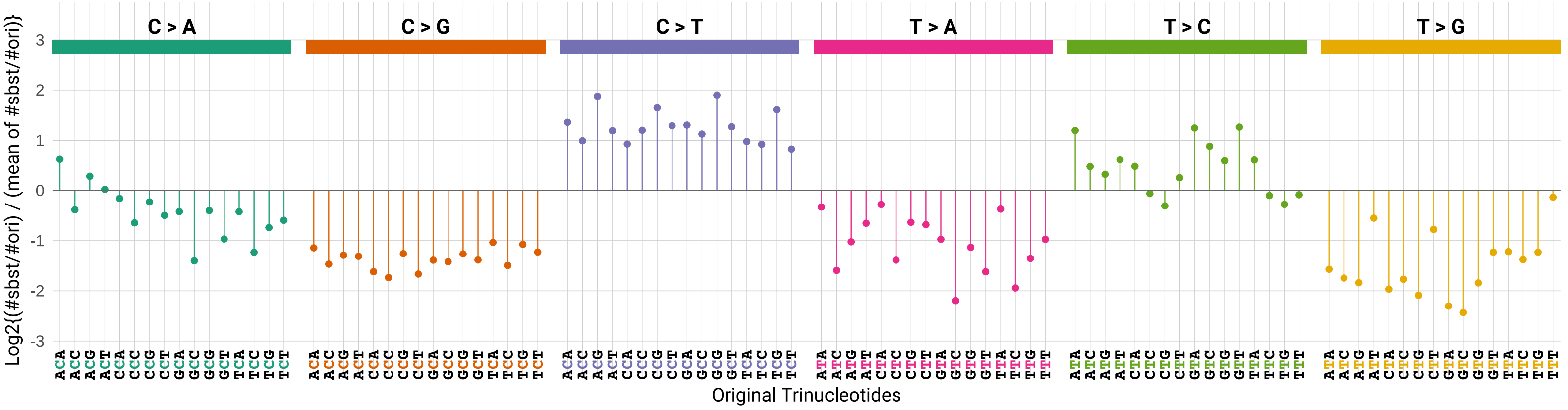

Montipora efflorescens

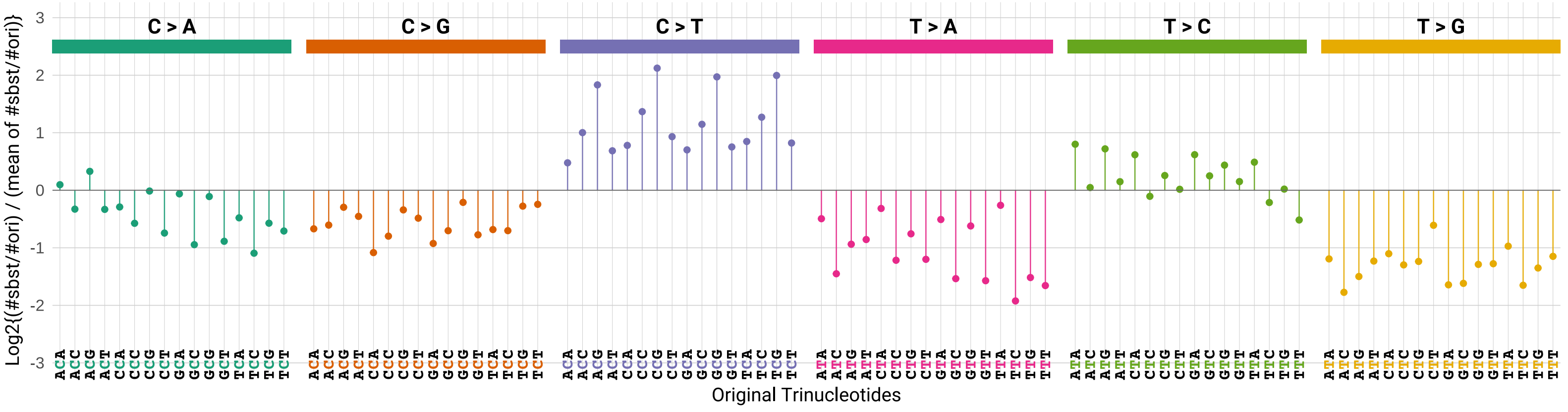

Supplementary Fig. S2.

Double-base substitution spectra for the fungi, *Podila humilis* and *Podila verticillata*. Both species show elevated CG>TT substitutions instead of the more typical double-base transition CG>TA. *P. verticillata* also shows elevated GC>AA substitutions rather than the more typical GC>AT.

Podila humilis

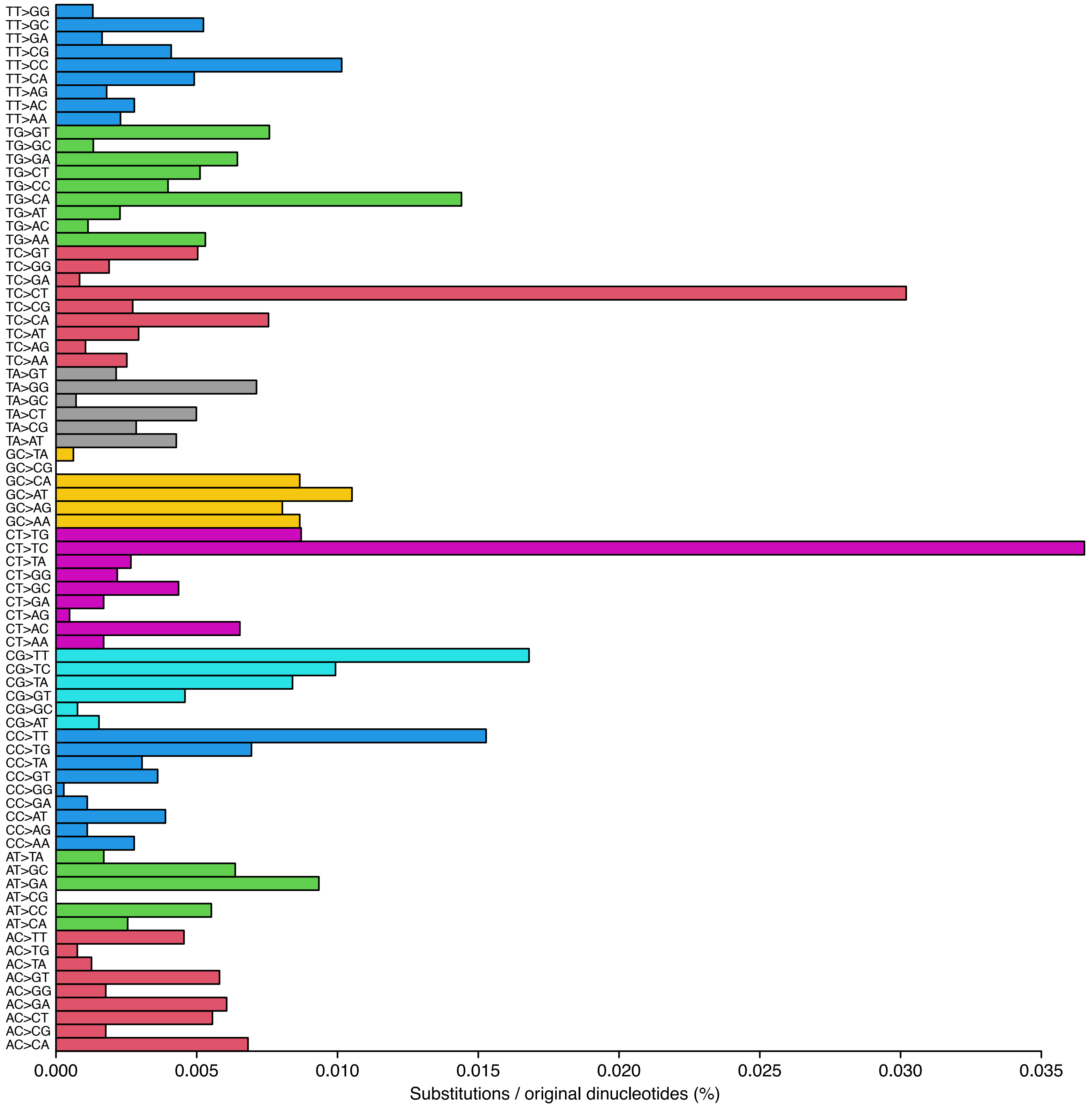

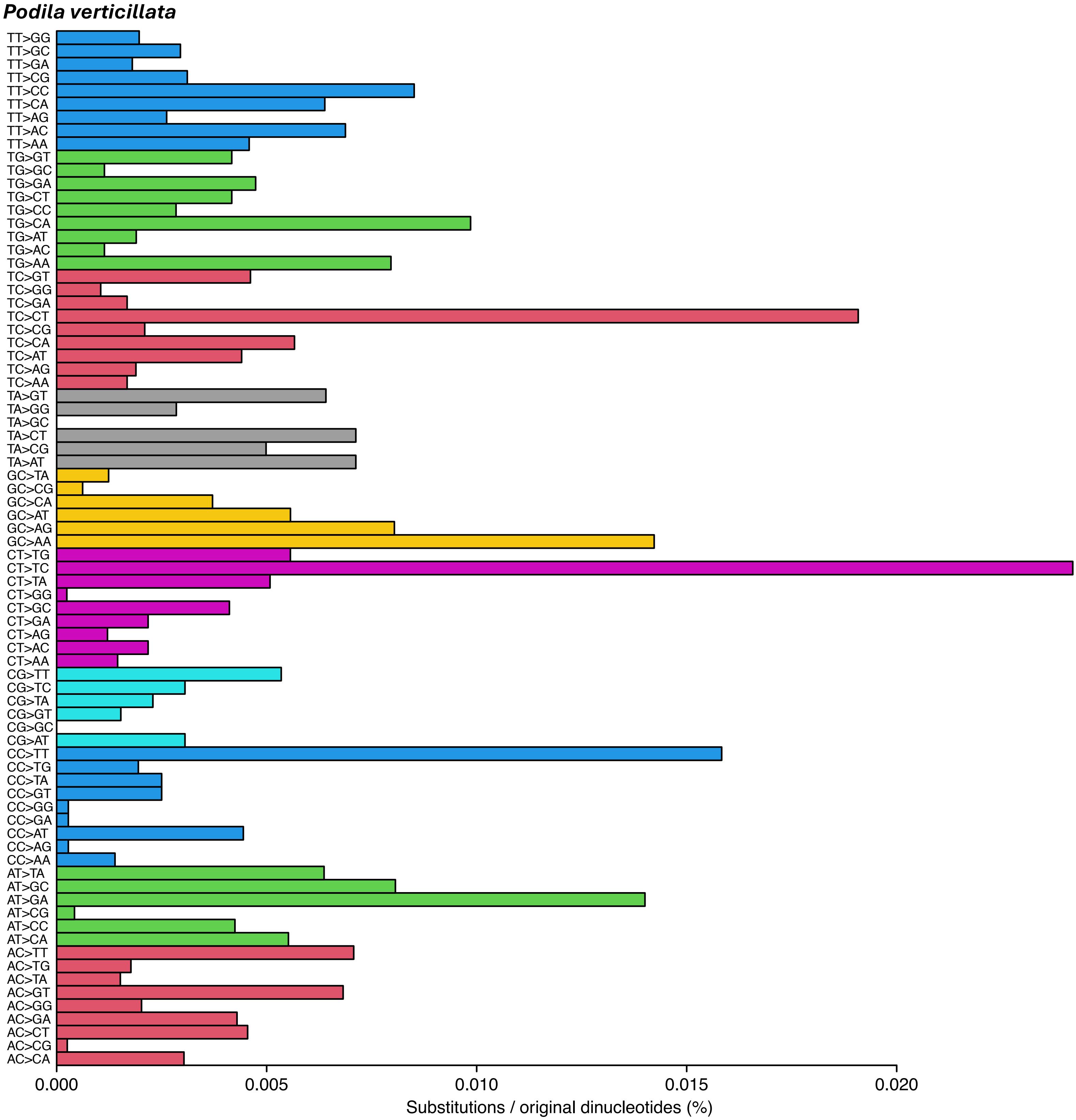

Podila verticillata
TT>GG
TT>GC
TT>GA
TT>CG
TT>CC
TT>CA
TT>AG
TT>AC
TT>AA
TG>GT
TG>GC
TG>GA
TG>CT
TG>CC
TG>CA
TG>AT
TG>AC
TG>AA
TC>GT
TC>GG
TC>GA
TC>CT
TC>CG
TC>CA
TC>AT
TC>AG
TC>AA
TA>GT
TA>GG
TA>GC
TA>CT
TA>CG
TA>AT
GC>TA
GC>CG
GC>CA
GC>AT
GC>AG
GC>AA
CT>TG
CT>TC
CT>TA
CT>GG
CT>GC
CT>GA
CT>AG
CT>AC
CT>AA
CG>TT
CG>TC
CG>TA
CG>GT
CG>GC
CG>AT
CC>TT
CC>TG
CC>TA
CC>GT
CC>GG
CC>GA
CC>AT
CC>AG
CC>AA
AT>TA
AT>GC
AT>GA
AT>CG
AT>CC
AT>CA
AC>TT
AC>TG
AC>TA
AC>GT
AC>GG
AC>GA
AC>CT
AC>CG
AC>CA
0.000
0.005
0.010
0.015
0.020
Substitutions / original dinucleotides (%)

Supplementary Table S1. Fungal TTA>TCA / TTA>TGA Enrichment

Fungal ingroups in which TTA>TCA or TTA>TGA ranks as the single trinucleotide context with the highest standardized Pearson residual within the T>C substitution category, or the T>G substitution category, respectively. Of the 54 fungal ingroups across 27 trios tested on the T>C category, 27 satisfied the top-single criterion; of the 54 ingroups tested on the T>G category, 29 satisfied the criterion. Notably, all ingroups satisfying the criterion belong to the class Agaricomycetes: 27 of the 36 Agaricomycetes ingroups (18 trios) for T>C, and 29 of 36 for T>G; no ingroup outside Agaricomycetes ranked TTA>TCA or TTA>TGA as the top context. The "Region" column indicates whether the analysis used non-coding regions only (nc) or the whole genome (wg).

* = $p < 0.05$, ** = $p < 0.01$

T>C CATEGORY — TOP CONTEXT TTA>TCA

Trio ID	Ingroup species	Region	p-value	Sig.	TTA>TCA stdres
Strpac1_Strluc2_Strste3	*Strobilurus luchuensis*	wg	$< 2.2 \times 10^{-308}$	**	89.99
Strpac1_Strluc2_Strste3	*Strobilurus stephanocystis*	wg	$< 2.2 \times 10^{-308}$	**	75.46
Lenedo1_Lenlat2_Lennov3	*Lentinula lateritia*	nc	$< 2.2 \times 10^{-308}$	**	57.84
Lacame1_Lacbic2_Lactri3	*Laccaria trichodermophora*	nc	$< 2.2 \times 10^{-308}$	**	54.95
Rusabi1_Rusgri2_Ruslep3	*Russula lepida*	wg	$< 2.2 \times 10^{-308}$	**	54.14
Lenedo1_Lenlat2_Lennov3	*Lentinula novae-zelandiae*	nc	$< 2.2 \times 10^{-308}$	**	52.73
Podmar1_Podpis2_Podrug3	*Podaxis rugospora*	wg	$< 2.2 \times 10^{-308}$	**	44.58
Lecobs1_Lecins2_Lecimi3	*Leccinum insolens*	wg	$< 2.2 \times 10^{-308}$	**	44.03
Bolrex1_Bolret2_Boledu3	*Boletus reticuloceps*	wg	$< 2.2 \times 10^{-308}$	**	42.15
Lecobs1_Lecins2_Lecimi3	*Leccinum imitatum*	wg	$< 2.2 \times 10^{-308}$	**	41.42
Bolrex1_Bolret2_Boledu3	*Boletus edulis*	wg	$< 2.2 \times 10^{-308}$	**	40.62
Lacame1_Lacbic2_Lactri3	*Laccaria bicolor*	nc	$< 2.2 \times 10^{-308}$	**	40.15
Bolbar1_Bolret2_Bolnob3	*Boletus reticulatus*	wg	$< 2.2 \times 10^{-308}$	**	38.89
Bolvar1_Boledu2_Bolrex3	*Boletus edulis*	wg	$< 2.2 \times 10^{-308}$	**	36.87
Armbor1_Armgal2_Armalt3	*Armillaria gallica*	nc	$< 2.2 \times 10^{-308}$	**	35.31
Bolbar1_Bolret2_Bolnob3	*Boletus nobilissimus*	wg	$< 2.2 \times 10^{-308}$	**	30.36
Lecglu1_Lecdis2_Lecpro3	*Leccinum disarticulatum*	wg	$< 2.2 \times 10^{-308}$	**	30.23
Bolvar1_Boledu2_Bolrex3	*Boletus rex-veris*	wg	$< 2.2 \times 10^{-308}$	**	29.73
Bolsem1_Boltyl2_Bolpse3	*Boletus pseudoseparans*	wg	$< 2.2 \times 10^{-308}$	**	28.75
Lecglu1_Lecdis2_Lecpro3	*Leccinum proximum*	wg	$< 2.2 \times 10^{-308}$	**	28.51
Pleost1_Pleery2_Pletuo3	*Pleurotus tuoliensis*	nc	$< 2.2 \times 10^{-308}$	**	28.47
Armbor1_Armgal2_Armalt3	*Armillaria altimontana*	nc	$< 2.2 \times 10^{-308}$	**	26.54
Inosue1_Inotig2_Inoflo3	*Inocybe flocculosa*	wg	$< 2.2 \times 10^{-308}$	**	24.10
Inosue1_Inotig2_Inoflo3	*Inocybe tigrina*	wg	$< 2.2 \times 10^{-308}$	**	23.67
Lacaka1_Lachen2_Lacpse3	*Lactarius hengduanensis*	nc	5.12×10^{-263}	**	18.60
Agabis1_Agabit2_Agasin3	*Agaricus bitorquis*	nc	1.80×10^{-87}	**	17.88
Agabis1_Agabit2_Agasin3	*Agaricus sinodeliciosus*	nc	2.57×10^{-50}	**	8.96

T>G CATEGORY — TOP CONTEXT TTA>TGA

Trio ID	Ingroup species	Region	p-value	Sig.	TTA>TGA stdres
Strpac1_Strluc2_Strste3	*Strobilurus luchuensis*	wg	$< 2.2 \times 10^{-308}$	**	48.52
Bolrex1_Bolret2_Boledu3	*Boletus reticuloceps*	wg	$< 2.2 \times 10^{-308}$	**	41.62
Lenedo1_Lenlat2_Lennov3	*Lentinula lateritia*	nc	$< 2.2 \times 10^{-308}$	**	41.18
Strpac1_Strluc2_Strste3	*Strobilurus stephanocystis*	wg	$< 2.2 \times 10^{-308}$	**	39.47
Bolrex1_Bolret2_Boledu3	*Boletus edulis*	wg	$< 2.2 \times 10^{-308}$	**	38.36
Lenedo1_Lenlat2_Lennov3	*Lentinula novae-zelandiae*	nc	$< 2.2 \times 10^{-308}$	**	38.04
Bolvar1_Boledu2_Bolrex3	*Boletus edulis*	wg	$< 2.2 \times 10^{-308}$	**	36.15
Podmar1_Podpis2_Podrug3	*Podaxis rugospora*	wg	$< 2.2 \times 10^{-308}$	**	31.91
Bolvar1_Boledu2_Bolrex3	*Boletus rex-veris*	wg	$< 2.2 \times 10^{-308}$	**	29.65
Bolbar1_Bolret2_Bolnob3	*Boletus reticulatus*	wg	$< 2.2 \times 10^{-308}$	**	27.03

Trio ID	Ingroup species	Region	p-value	Sig.	TTA>TGA stdres
Lecobs1_Lecins2_Lecimi3	*Leccinum imitatum*	wg	1.11×10^{-240}	**	26.36
Lecobs1_Lecins2_Lecimi3	*Leccinum insolens*	wg	2.77×10^{-219}	**	24.32
Bolsem1_Boltyl2_Bolpse3	*Boletus pseudoseparans*	wg	1.14×10^{-259}	**	23.46
Lacame1_Lacbic2_Lactri3	*Laccaria trichodermophora*	nc	8.89×10^{-189}	**	22.57
Bolbar1_Bolret2_Bolnob3	*Boletus nobilissimus*	wg	8.12×10^{-221}	**	20.84
Bolsem1_Boltyl2_Bolpse3	*Boletus tylopilopsis*	wg	9.61×10^{-255}	**	19.97
Lecglu1_Lecdis2_Lecpro3	*Leccinum disarticulatum*	wg	5.28×10^{-169}	**	18.71
Armbor1_Armgal2_Armalt3	*Armillaria gallica*	nc	3.23×10^{-197}	**	18.33
Lacame1_Lacbic2_Lactri3	*Laccaria bicolor*	nc	4.66×10^{-123}	**	18.06
Lecglu1_Lecdis2_Lecpro3	*Leccinum proximum*	wg	1.05×10^{-146}	**	17.82
Pleost1_Pleery2_Pletuo3	*Pleurotus tuoliensis*	nc	9.02×10^{-143}	**	16.87
Lacsan1_Lacdel2_Lachat3	*Lactarius hatsudake*	nc	2.98×10^{-184}	**	13.95
Bolnan1_Boltom2_Bolmar3	*Boletus tomentosulus*	wg	2.40×10^{-71}	**	13.03
Inosue1_Inotig2_Inoflo3	*Inocybe flocculosa*	wg	7.21×10^{-142}	**	12.91
Armbor1_Armgal2_Armalt3	*Armillaria altimontana*	nc	9.64×10^{-154}	**	12.50
Pleost1_Pleery2_Pletuo3	*Pleurotus eryngii*	nc	2.10×10^{-77}	**	11.28
Bolnan1_Boltom2_Bolmar3	*Boletus mariae*	wg	1.35×10^{-60}	**	10.74
Lacaka1_Lachen2_Lacpse3	*Lactarius hengduanensis*	nc	1.30×10^{-97}	**	9.71
Agabis1_Agabit2_Agasin3	*Agaricus bitorquis*	nc	1.74×10^{-11}	**	4.69

Supplementary Table S2. Cnidarian ACA>AAA / ACG>AAG Enrichment

Cnidarian ingroups in which ACA>AAA and ACG>AAG rank as the top two trinucleotide contexts by standardized Pearson residual within the C>A substitution category. All reported p-values fell below the minimum representable double-precision floating-point number and are shown as $< 2.2 \times 10^{-308}$. Of the 32 cnidarian ingroups across 16 trios for which the test could be run on the C>A category, 31 satisfied the top-two criterion and are shown below. The "Region" column indicates whether the analysis used non-coding regions only (nc) or the whole genome (wg).

** = p < 0.01

Trio ID	Ingroup species	Region	p-value	Sig.	ACA>AAA stdres	ACG>AAG stdres
Porrus1_Porlob2_Porlut3	*Porites lobata*	wg	$< 2.2 \times 10^{-308}$	**	128.28	156.83
Acraus1_Acrten2_Acrspa3	*Acropora spathulata*	wg	$< 2.2 \times 10^{-308}$	**	118.31	114.98
Pocgra1_Pocver2_Pocdam3	*Pocillopora verrucosa*	wg	$< 2.2 \times 10^{-308}$	**	115.68	61.42
Porrus1_Porlob2_Porlut3	*Porites lutea*	wg	$< 2.2 \times 10^{-308}$	**	111.65	93.46
Echhor1_Orbfav2_Cypsal3	*Orbicella faveolata*	wg	$< 2.2 \times 10^{-308}$	**	90.74	62.23
Echhor1_Orbfav2_Cypsal3	*Cyphastrea salae*	wg	$< 2.2 \times 10^{-308}$	**	87.74	86.12
Pocgra1_Pocver2_Pocdam3	*Pocillopora damicornis*	wg	$< 2.2 \times 10^{-308}$	**	84.70	39.07
Acraus1_Acrten2_Acrspa3	*Acropora tenuis*	wg	$< 2.2 \times 10^{-308}$	**	80.65	63.03
Porrus1_Poraus2_Porcyl3	*Porites cylindrica*	wg	$< 2.2 \times 10^{-308}$	**	75.33	88.15
Porrus1_Poraus2_Porcyl3	*Porites australiensis*	wg	$< 2.2 \times 10^{-308}$	**	72.90	85.05
Palcar1_Palmut2_Palgra3	*Palythoa grandiflora*	wg	$< 2.2 \times 10^{-308}$	**	72.75	39.94
Palcar1_Palmut2_Palgra3	*Palythoa mutuki*	wg	$< 2.2 \times 10^{-308}$	**	67.24	42.05
Casorn1_Casxam2_Casand3	*Cassiopea xamachana*	wg	$< 2.2 \times 10^{-308}$	**	66.73	17.37
Hetmag1_Stihad2_Stimer3	*Stichodactyla haddoni*	wg	$< 2.2 \times 10^{-308}$	**	66.34	34.59
Acrmil1_Acrnas2_Acrmic3	*Acropora nasuta*	nc	$< 2.2 \times 10^{-308}$	**	66.16	56.82
Casorn1_Casxam2_Casand3	*Cassiopea andromeda*	wg	$< 2.2 \times 10^{-308}$	**	65.73	16.27
Moncap1_Mongri2_Moneff3	*Montipora efflorescens*	wg	$< 2.2 \times 10^{-308}$	**	63.61	64.18
Stypis1_Pocver2_Pocdam3	*Pocillopora verrucosa*	nc	$< 2.2 \times 10^{-308}$	**	63.52	13.95
Acrdig1_Acrhya2_Acrmil3	*Acropora hyacinthus*	nc	$< 2.2 \times 10^{-308}$	**	63.10	54.41
Acrmil1_Acrnas2_Acrmic3	*Acropora microphthalma*	nc	$< 2.2 \times 10^{-308}$	**	62.76	55.67
Stypis1_Pocver2_Pocdam3	*Pocillopora damicornis*	nc	$< 2.2 \times 10^{-308}$	**	62.50	15.93
Moncap1_Mongri2_Moneff3	*Montipora grisea*	wg	$< 2.2 \times 10^{-308}$	**	61.70	64.37
Hetmag1_Stihad2_Stimer3	*Stichodactyla mertensii*	wg	$< 2.2 \times 10^{-308}$	**	58.42	38.23

Trio ID	Ingroup species	Region	p-value	Sig.	ACA>AAA stdres	ACG>AAG stdres
Acrdig1_Acrhya2_Acrmil3	*Acropora millepora*	nc	$< 2.2 \times 10^{-308}$	**	58.01	51.66
Dunaxi1_Dencri2_Tubcoc3	*Tubastraea coccinea*	wg	$< 2.2 \times 10^{-308}$	**	57.76	30.60
Acrdig1_Acrhya2_Acrspi3	*Acropora spicifera*	nc	$< 2.2 \times 10^{-308}$	**	56.93	49.36
Acrdig1_Acrhya2_Acrspi3	*Acropora hyacinthus*	nc	$< 2.2 \times 10^{-308}$	**	54.89	49.93
Dunaxi1_Dencri2_Tubcoc3	*Dendrophyllia cribrosa*	wg	$< 2.2 \times 10^{-308}$	**	45.98	22.31
Cypsal1_Orbfav2_Orbfra3	*Orbicella franksi*	wg	$< 2.2 \times 10^{-308}$	**	43.09	27.92
Cypsal1_Orbfav2_Orbfra3	*Orbicella faveolata*	wg	$< 2.2 \times 10^{-308}$	**	39.79	26.16
Palmiz1_Palcar2_Palmut3	*Palythoa caribaeorum*	nc	$< 2.2 \times 10^{-308}$	**	30.27	10.66

Supplementary Table S3. Statistical tests of non-uniform dinucleotide substitutions for CG and GC substitutions in *Podila humilis* and *Podila verticillata*.

For each ancestral dinucleotide (origDblt), we tested whether substitution counts were uniformly distributed across all possible mutated targets, using either a chi-square test or Fisher's exact test when expected counts were small (≥ 5). Results are shown for both non-coding and whole-genome datasets. For each substitution context, the observed count, expected count under the null hypothesis, and standardized residual (stdres) are reported.

* $p < 0.05$, ** $p < 0.01$

PODILA HUMILIS NON-CODING REGION

origDblt	method	statistic	df	*p*-value	sig
CG	chisq	33.9115	5	2.4796e-06	**

context	observed	expected	stdres
CG>AT	2	9.17	-2.59
CG>GC	1	9.17	-2.95
CG>GT	6	9.17	-1.15
CG>TA	11	9.17	0.66
CG>TC	13	9.17	1.39
CG>TT	22	9.17	4.64

PODILA HUMILIS WHOLE GENOME

origDblt	method	statistic	df	p-value	sig
CG	chisq	34.4623	5	1.9260e-06	**

context	observed	expected	stdres
CG>AT	4	13.00	-2.73
CG>GC	2	13.00	-3.34
CG>GT	10	13.00	-0.91
CG>TA	18	13.00	1.52
CG>TC	17	13.00	1.22
CG>TT	27	13.00	4.25

PODILA VERTICILLATA NON-CODING REGION

origDblt	method	statistic	df	*p*-value	sig
CG	fisher	NA	NA	1.1539e-01	

context	observed	expected	stdres
CG>AT	4	3.33	0.40
CG>GC	0	3.33	-2.00
CG>GT	2	3.33	-0.80
CG>TA	3	3.33	-0.20
CG>TC	4	3.33	0.40
CG>TT	7	3.33	2.20

origDblt	method	statistic	df	*p*-value	sig
GC	chisq	37.1132	5	5.6846e-07 **	

context	observed	expected	stdres
GC>AA	23	9.00	5.11
GC>AG	13	9.00	1.46
GC>AT	9	9.00	0.00
GC>CA	6	9.00	-1.10
GC>CG	1	9.00	-2.92
GC>TA	2	9.00	-2.56

PODILA VERTICILLATA WHOLE GENOME

origDblt	method	statistic	df	*p*-value	sig
CG	chisq	17.2859	5	3.9883e-03	**

context	observed	expected	stdres
CG>AT	4	5.83	-0.83
CG>GC	0	5.83	-2.65
CG>GT	3	5.83	-1.29
CG>TA	10	5.83	1.89
CG>TC	6	5.83	0.08
CG>TT	12	5.83	2.80

origDblt	method	statistic	df	*p*-value	sig
GC	chisq	51.8618	5	5.7586e-10	**

context	observed	expected	stdres
GC>AA	32	13.17	5.69
GC>AG	18	13.17	1.46
GC>AT	17	13.17	1.16
GC>CA	9	13.17	-1.26
GC>CG	1	13.17	-3.67
GC>TA	2	13.17	-3.37

Supplementary Table S4. Glomeromycetes A:T-rich Dinucleotide Substitution Enrichment

Glomeromycetes ingroups in which the specified A:T-rich doublet substitution (TT>AA, TA>AT, or AT>TA) ranks first by standardized Pearson residual within its original doublet category. "Region" indicates whether the analysis used non-coding regions only (nc) or whole-genome alignment (wg).

* = p < 0.05, ** = p < 0.01

TT > AA ENRICHMENT

Trio ID	Ingroup species	Region	p-value	Sig.	TT>AA stdres
Fungeo1_Funcal2_Funmos3	*Funneliformis caledonium*	nc	1.15×10^{-154}	**	23.81
Fungeo1_Funcal2_Funmos3	*Funneliformis caledonium*	wg	9.81×10^{-152}	**	23.18
Fungeo1_Funcal2_Funmos3	*Funneliformis mosseae*	nc	9.60×10^{-125}	**	20.63
Fungeo1_Funcal2_Funmos3	*Funneliformis mosseae*	wg	1.88×10^{-128}	**	20.34
Denhet1_Gigros2_Gigmar3	*Gigaspora margarita*	nc	1.86×10^{-184}	**	18.77
Denhet1_Gigros2_Gigmar3	*Gigaspora margarita*	wg	2.13×10^{-211}	**	18.21
Rhipro1_Rhiirr2_Rhiirr3	*Rhizophagus irregularis* (GCA_020716745.1)	wg	8.73×10^{-86}	**	15.68
Rhipro1_Rhiirr2_Rhiirr3	*Rhizophagus irregularis* (GCF_026210795.1)	wg	4.11×10^{-69}	**	12.38

TA > AT ENRICHMENT

Trio ID	Ingroup species	Region	p-value	Sig.	TA>AT stdres
Fungeo1_Funcal2_Funmos3	*Funneliformis caledonium*	nc	4.14×10^{-92}	**	20.02

Trio ID	Ingroup species	Region	p-value	Sig.	TA>AT stdres
Fungeo1_Funcal2_Funmos3	*Funneliformis caledonium*	wg	1.09×10^{-88}	**	19.39
Fungeo1_Funcal2_Funmos3	*Funneliformis mosseae*	nc	2.37×10^{-63}	**	15.81
Fungeo1_Funcal2_Funmos3	*Funneliformis mosseae*	wg	1.05×10^{-61}	**	15.37
Denhet1_Gigros2_Gigmar3	*Gigaspora margarita*	nc	8.19×10^{-62}	**	10.32
Denhet1_Gigros2_Gigmar3	*Gigaspora rosea*	nc	7.95×10^{-76}	**	11.17
Denhet1_Gigros2_Gigmar3	*Gigaspora rosea*	wg	1.05×10^{-82}	**	9.76
Rhipro1_Rhiirr2_Rhiirr3	*Rhizophagus irregularis* (GCA_020716745.1)	wg	1.18×10^{-40}	**	11.62
Rhicla1_Rhiirr2_Rhipro3	*Rhizophagus irregularis* (GCF_026210795.1)	nc	1.99×10^{-106}	**	12.17
Rhipro1_Rhiirr2_Rhiirr3	Rhizophagus irregularis (GCF_026210795.1)	wg	3.52×10^{-11}	**	3.42

AT > TA ENRICHMENT

Trio ID	Ingroup species	Region	p-value	Sig.	AT>TA stdres
Fungeo1_Funcal2_Funmos3	*Funneliformis mosseae*	nc	1.60×10^{-68}	**	16.59
Fungeo1_Funcal2_Funmos3	*Funneliformis mosseae*	wg	2.72×10^{-63}	**	15.53
Fungeo1_Funcal2_Funmos3	*Funneliformis caledonium*	nc	2.96×10^{-62}	**	15.34
Fungeo1_Funcal2_Funmos3	*Funneliformis caledonium*	wg	3.14×10^{-60}	**	14.81
Denhet1_Gigros2_Gigmar3	*Gigaspora margarita*	nc	5.90×10^{-56}	**	8.68
Denhet1_Gigros2_Gigmar3	*Gigaspora rosea*	nc	1.06×10^{-74}	**	10.18
Rhipro1_Rhiirr2_Rhiirr3	*Rhizophagus irregularis* (GCA_020716745.1)	wg	2.16×10^{-43}	**	10.98

Supplementary Table S5. Fungi trios

All fungal trios investigated in this study.

Trio ID	Role	Organism name	Accession	Kingdom	Phylum	Class	Order	Family
**Agabis1_Agabit2_Agasin3** *[Non-coding: Yes]*	outgroup	*Agaricus bisporus var. bisporus H97*	GCF_000300575.1	Fungi	Basidiomycota	Agaricomycetes	Agaricales	Agaricaceae
	ingroup	*Agaricus bitorquis*	GCA_030246685.1	Fungi	Basidiomycota	Agaricomycetes	Agaricales	Agaricaceae
	ingroup	*Agaricus sinodeliciosus*	GCA_022315185.1	Fungi	Basidiomycota	Agaricomycetes	Agaricales	Agaricaceae
**Armbor1_Armgal2_Armalt3** *[Non-coding: Yes]*	outgroup	*Armillaria borealis*	GCA_030435635.1	Fungi	Basidiomycota	Agaricomycetes	Agaricales	Physalacriaceae
	ingroup	*Armillaria gallica*	GCA_037576215.1	Fungi	Basidiomycota	Agaricomycetes	Agaricales	Physalacriaceae
	ingroup	*Armillaria altimontana*	GCA_022818075.1	Fungi	Basidiomycota	Agaricomycetes	Agaricales	Physalacriaceae
**Bolbar1_Bolret2_Bolnob3** *[Non-coding: No]*	outgroup	*Boletus barrowsii*	GCA_038088775.1	Fungi	Basidiomycota	Agaricomycetes	Boletales	Boletaceae

Trio ID	Role	Organism name	Accession	Kingdom	Phylum	Class	Order	Family
	ingroup	*Boletus reticulatus*	GCA_038093535.1	Fungi	Basidiomycota	Agaricomycetes	Boletales	Boletaceae
	ingroup	*Boletus nobilissimus*	GCA_038088815.1	Fungi	Basidiomycota	Agaricomycetes	Boletales	Boletaceae
**Bolnan1_Boltom2_Bolmar3** *[Non-coding: No]*	outgroup	*Boletus nancyae*	GCA_038093035.1	Fungi	Basidiomycota	Agaricomycetes	Boletales	Boletaceae
	ingroup	*Boletus tomentosulus*	GCA_038092475.1	Fungi	Basidiomycota	Agaricomycetes	Boletales	Boletaceae
	ingroup	*Boletus mariae*	GCA_038093055.1	Fungi	Basidiomycota	Agaricomycetes	Boletales	Boletaceae
**Bolrex1_Bolret2_Boledu3** *[Non-coding: No]*	outgroup	*Boletus rex-veris*	GCA_038088795.1	Fungi	Basidiomycota	Agaricomycetes	Boletales	Boletaceae
	ingroup	*Boletus reticuloceps*	GCA_018397855.1	Fungi	Basidiomycota	Agaricomycetes	Boletales	Boletaceae
	ingroup	*Boletus edulis BED1*	GCA_015179015.1	Fungi	Basidiomycota	Agaricomycetes	Boletales	Boletaceae
**Bolsem1_Boltyl2_Bolpse3** *[Non-coding: No]*	outgroup	*Boletus semigastroideus*	GCA_038090295.1	Fungi	Basidiomycota	Agaricomycetes	Boletales	Boletaceae
	ingroup	*Boletus tylopilopsis*	GCA_038093875.1	Fungi	Basidiomycota	Agaricomycetes	Boletales	Boletaceae
	ingroup	*Boletus pseudoseparans*	GCA_038092835.1	Fungi	Basidiomycota	Agaricomycetes	Boletales	Boletaceae
**Bolvar1_Boledu2_Bolrex3** *[Non-coding: No]*	outgroup	*Boletus variipes var. fagicola*	GCA_038092395.1	Fungi	Basidiomycota	Agaricomycetes	Boletales	Boletaceae
	ingroup	*Boletus edulis BED1*	GCA_015179015.1	Fungi	Basidiomycota	Agaricomycetes	Boletales	Boletaceae
	ingroup	*Boletus rex-veris*	GCA_038088795.1	Fungi	Basidiomycota	Agaricomycetes	Boletales	Boletaceae
**Inosue1_Inotig2_Inoflo3** *[Non-coding: No]*	outgroup	*Inocybe suecica*	GCA_043168805.1	Fungi	Basidiomycota	Agaricomycetes	Agaricales	Inocybaceae
	ingroup	*Inocybe tigrina*	GCA_964248975.1	Fungi	Basidiomycota	Agaricomycetes	Agaricales	Inocybaceae
	ingroup	*Inocybe flocculosa*	GCA_043167125.1	Fungi	Basidiomycota	Agaricomycetes	Agaricales	Inocybaceae
**Lacaka1_Lachen2_Lacpse3** *[Non-coding: Yes]*	outgroup	*Lactarius akahatsu*	GCA_021524915.1	Fungi	Basidiomycota	Agaricomycetes	Russulales	Russulaceae

Trio ID	Role	Organism name	Accession	Kingdom	Phylum	Class	Order	Family
	ingroup	*Lactarius hengduanensis*	GCA_021525025.1	Fungi	Basidiomycota	Agaricomycetes	Russulales	Russulaceae
	ingroup	*Lactarius pseudohatsudake*	GCA_021525015.1	Fungi	Basidiomycota	Agaricomycetes	Russulales	Russulaceae
**Lacame1_Lacbic2_Lactri3** *[Non-coding: Yes]*	outgroup	*Laccaria amethystina LaAM-08-1*	GCA_000827195.1	Fungi	Basidiomycota	Agaricomycetes	Agaricales	Hydnangiaceae
	ingroup	*Laccaria bicolor S238N-H82*	GCF_000143565.1	Fungi	Basidiomycota	Agaricomycetes	Agaricales	Hydnangiaceae
	ingroup	*Laccaria trichodermophora*	GCA_018417955.1	Fungi	Basidiomycota	Agaricomycetes	Agaricales	Hydnangiaceae
**Lacsan1_Lacdel2_Lachat3** *[Non-coding: Yes]*	outgroup	*Lactarius sanguifluus*	GCA_021527775.1	Fungi	Basidiomycota	Agaricomycetes	Russulales	Russulaceae
	ingroup	*Lactarius deliciosus*	GCA_021525775.1	Fungi	Basidiomycota	Agaricomycetes	Russulales	Russulaceae
	ingroup	*Lactarius hatsudake*	GCA_024734325.1	Fungi	Basidiomycota	Agaricomycetes	Russulales	Russulaceae
**Lecglu1_Lecdis2_Lecpro3** *[Non-coding: No]*	outgroup	*Leccinum glutinopallens*	GCA_038092055.1	Fungi	Basidiomycota	Agaricomycetes	Boletales	Boletaceae
	ingroup	*Leccinum disarticulatum*	GCA_038092035.1	Fungi	Basidiomycota	Agaricomycetes	Boletales	Boletaceae
	ingroup	*Leccinum proximum*	GCA_038091695.1	Fungi	Basidiomycota	Agaricomycetes	Boletales	Boletaceae
**Lecobs1_Lecins2_Lecimi3** *[Non-coding: No]*	outgroup	*Leccinum obscurum*	GCA_038091815.1	Fungi	Basidiomycota	Agaricomycetes	Boletales	Boletaceae
	ingroup	*Leccinum insolens*	GCA_038091875.1	Fungi	Basidiomycota	Agaricomycetes	Boletales	Boletaceae
	ingroup	*Leccinum imitatum*	GCA_038091955.1	Fungi	Basidiomycota	Agaricomycetes	Boletales	Boletaceae
**Lenedo1_Lenlat2_Lennov3** *[Non-coding: Yes]*	outgroup	*Lentinula edodes*	GCF_021015755.1	Fungi	Basidiomycota	Agaricomycetes	Agaricales	Omphalotaceae
	ingroup	*Lentinula lateritia*	GCA_028011325.1	Fungi	Basidiomycota	Agaricomycetes	Agaricales	Omphalotaceae
	ingroup	*Lentinula novae-zelandiae*	GCA_027921425.1	Fungi	Basidiomycota	Agaricomycetes	Agaricales	Omphalotaceae
**Pleost1_Pleery2_Pletuo3** *[Non-coding: Yes]*	outgroup	*Pleurotus ostreatus*	GCF_014466165.1	Fungi	Basidiomycota	Agaricomycetes	Agaricales	Pleurotaceae

Trio ID	Role	Organism name	Accession	Kingdom	Phylum	Class	Order	Family
	ingroup	*Pleurotus eryngii var. eryngii*	GCA_029467805.1	Fungi	Basidiomycota	Agaricomycetes	Agaricales	Pleurotaceae
	ingroup	*Pleurotus tuoliensis*	GCA_036872985.1	Fungi	Basidiomycota	Agaricomycetes	Agaricales	Pleurotaceae
**Podmar1_Podpis2_Podrug3** *[Non-coding: No]*	outgroup	*Podaxis mareebaensis*	GCA_018524725.1	Fungi	Basidiomycota	Agaricomycetes	Agaricales	Agaricaceae
	ingroup	*Podaxis pistillaris*	GCA_018524465.1	Fungi	Basidiomycota	Agaricomycetes	Agaricales	Agaricaceae
	ingroup	*Podaxis rugospora*	GCA_018524415.1	Fungi	Basidiomycota	Agaricomycetes	Agaricales	Agaricaceae
**Rusabi1_Rusgri2_Ruslep3** *[Non-coding: No]*	outgroup	*Russula abietina*	GCA_003313715.1	Fungi	Basidiomycota	Agaricomycetes	Russulales	Russulaceae
	ingroup	*Russula griseocarnosa*	GCA_022884055.1	Fungi	Basidiomycota	Agaricomycetes	Russulales	Russulaceae
	ingroup	*Russula lepida*	GCA_003316425.1	Fungi	Basidiomycota	Agaricomycetes	Russulales	Russulaceae
**Strpac1_Strluc2_Strste3** *[Non-coding: No]*	outgroup	*Strobilurus pachycystidiatus*	GCA_019915135.1	Fungi	Basidiomycota	Agaricomycetes	Agaricales	Physalacriaceae
	ingroup	*Strobilurus luchuensis*	GCA_019915105.1	Fungi	Basidiomycota	Agaricomycetes	Agaricales	Physalacriaceae
	ingroup	*Strobilurus stephanocystis*	GCA_019915075.1	Fungi	Basidiomycota	Agaricomycetes	Agaricales	Physalacriaceae
**Podmin1_Podver2_Podhum3** *[Non-coding: Yes]*	outgroup	*Podila minutissima*	GCA_016098005.1	Fungi	Mucoromycota	Mortierellomycetes	Mortierellales	Mortierellaceae
	ingroup	*Podila verticillata NRRL 6337*	GCA_000739165.1	Fungi	Mucoromycota	Mortierellomycetes	Mortierellales	Mortierellaceae
	ingroup	*Podila humilis*	GCA_025677895.1	Fungi	Mucoromycota	Mortierellomycetes	Mortierellales	Mortierellaceae
**Denhet1_Gigros2_Gigmar3** *[Non-coding: Yes]*	outgroup	*Dentiscutata heterogama*	GCA_910591775.1	Fungi	Mucoromycota	Glomeromycetes	Diversisporales	Gigasporaceae
	ingroup	*Gigaspora rosea*	GCA_003550325.1	Fungi	Mucoromycota	Glomeromycetes	Diversisporales	Gigasporaceae
	ingroup	*Gigaspora margarita*	GCA_009809945.1	Fungi	Mucoromycota	Glomeromycetes	Diversisporales	Gigasporaceae
**Fungeo1_Funcal2_Funmos3** *[Non-coding: Yes]*	outgroup	*Funneliformis geosporum*	GCA_946474995.1	Fungi	Mucoromycota	Glomeromycetes	Glomerales	Glomeraceae
	ingroup	*Funneliformis caledonium*	GCA_910591825.1	Fungi	Mucoromycota	Glomeromycetes	Glomerales	Glomeraceae

Trio ID	Role	Organism name	Accession	Kingdom	Phylum	Class	Order	Family
	ingroup	*Funneliformis mosseae*	GCA_910592005.1	Fungi	Mucoromycota	Glomeromycetes	Glomerales	Glomeraceae
**Parbra1_Parocc2_Parocc3** *[Non-coding: Yes]*	outgroup	*Paraglomus brasilianum*	GCA_910592345.1	Fungi	Mucoromycota	Glomeromycetes	Paraglomerales	Paraglomeraceae
	ingroup	*Paraglomus occultum*	GCA_910592205.1	Fungi	Mucoromycota	Glomeromycetes	Paraglomerales	Paraglomeraceae
	ingroup	*Paraglomus occultum*	GCA_022605545.1	Fungi	Mucoromycota	Glomeromycetes	Paraglomerales	Paraglomeraceae
**Rhicla1_Rhiirr2_Rhipro3** *[Non-coding: Yes]*	outgroup	*Rhizophagus clarus*	GCA_015698045.1	Fungi	Mucoromycota	Glomeromycetes	Glomerales	Glomeraceae
	ingroup	*Rhizophagus irregularis*	GCF_026210795.1	Fungi	Mucoromycota	Glomeromycetes	Glomerales	Glomeraceae
	ingroup	*Rhizophagus prolifer*	GCA_019425655.1	Fungi	Mucoromycota	Glomeromycetes	Glomerales	Glomeraceae
**Rhipro1_Rhiirr2_Rhiirr3** *[Non-coding: No]*	outgroup	*Rhizophagus prolifer*	GCA_019425655.1	Fungi	Mucoromycota	Glomeromycetes	Glomerales	Glomeraceae
	ingroup	*Rhizophagus irregularis*	GCF_026210795.1	Fungi	Mucoromycota	Glomeromycetes	Glomerales	Glomeraceae
	ingroup	*Rhizophagus irregularis*	GCA_020716745.1	Fungi	Mucoromycota	Glomeromycetes	Glomerales	Glomeraceae

Supplementary Table S6. Cnidaria trios

All cnidarian trios investigated in this study.

Trio ID	Role	Organism name	Accession	Kingdom	Phylum	Class	Order	Family
**Acraus1_Acrten2_Acrspa3** *[Non-coding: No]*	outgroup	*Acropora austera*	GCA_964273435.1	Metazoa	Cnidaria	Anthozoa	Scleractinia	Acroporidae
	ingroup	*Acropora tenuis*	GCA_014633955.1	Metazoa	Cnidaria	Anthozoa	Scleractinia	Acroporidae
	ingroup	*Acropora spathulata*	GCA_031770025.1	Metazoa	Cnidaria	Anthozoa	Scleractinia	Acroporidae
**Acrdig1_Acrhya2_Acrmil3** *[Non-coding: Yes]*	outgroup	*Acropora digitifera*	GCF_000222465.1	Metazoa	Cnidaria	Anthozoa	Scleractinia	Acroporidae
	ingroup	*Acropora hyacinthus*	GCA_964291705.1	Metazoa	Cnidaria	Anthozoa	Scleractinia	Acroporidae
	ingroup	*Acropora millepora*	GCF_013753865.1	Metazoa	Cnidaria	Anthozoa	Scleractinia	Acroporidae
**Acrdig1_Acrhya2_Acrspi3** *[Non-coding: Yes]*	outgroup	*Acropora digitifera*	GCF_000222465.1	Metazoa	Cnidaria	Anthozoa	Scleractinia	Acroporidae

Trio ID	Role	Organism name	Accession	Kingdom	Phylum	Class	Order	Family
	ingroup	*Acropora hyacinthus*	GCA_964291705.1	Metazoa	Cnidaria	Anthozoa	Scleractinia	Acroporidae
	ingroup	*Acropora spicifera*	GCA_964261235.1	Metazoa	Cnidaria	Anthozoa	Scleractinia	Acroporidae
**Acrmil1_Acrnas2_Acrmic3** *[Non-coding: Yes]*	outgroup	*Acropora millepora*	GCF_013753865.1	Metazoa	Cnidaria	Anthozoa	Scleractinia	Acroporidae
	ingroup	*Acropora nasuta*	GCA_014634205.1	Metazoa	Cnidaria	Anthozoa	Scleractinia	Acroporidae
	ingroup	*Acropora microphthalma*	GCA_014634165.1	Metazoa	Cnidaria	Anthozoa	Scleractinia	Acroporidae
**Casorn1_Casxam2_Casand3** *[Non-coding: No]*	outgroup	*Cassiopea ornata*	GCA_964304725.1	Metazoa	Cnidaria	Scyphozoa	Rhizostomeae	Cassiopeidae
	ingroup	*Cassiopea xamachana*	GCA_964235115.1	Metazoa	Cnidaria	Scyphozoa	Rhizostomeae	Cassiopeidae
	ingroup	*Cassiopea andromeda*	GCA_018155075.1	Metazoa	Cnidaria	Scyphozoa	Rhizostomeae	Cassiopeidae
**Cypsal1_Orbfav2_Orbfra3** *[Non-coding: No]*	outgroup	*Cyphastrea salae*	GCA_964194085.1	Metazoa	Cnidaria	Anthozoa	Scleractinia	Merulinidae
	ingroup	*Orbicella faveolata*	GCF_002042975.1	Metazoa	Cnidaria	Anthozoa	Scleractinia	Merulinidae
	ingroup	*Orbicella franksi*	GCA_964199315.1	Metazoa	Cnidaria	Anthozoa	Scleractinia	Merulinidae
**Dunaxi1_Dencri2_Tubcoc3** *[Non-coding: No]*	outgroup	*Duncanopsammia axifuga*	GCA_964258685.1	Metazoa	Cnidaria	Anthozoa	Scleractinia	Dendrophylliidae
	ingroup	*Dendrophyllia cribrosa*	GCA_024195265.1	Metazoa	Cnidaria	Anthozoa	Scleractinia	Dendrophylliidae
	ingroup	*Tubastraea coccinea*	GCA_047759845.1	Metazoa	Cnidaria	Anthozoa	Scleractinia	Dendrophylliidae
**Echhor1_Orbfav2_Cypsal3** *[Non-coding: No]*	outgroup	*Echinopora horrida*	GCA_964199735.2	Metazoa	Cnidaria	Anthozoa	Scleractinia	Merulinidae
	ingroup	*Orbicella faveolata*	GCF_002042975.1	Metazoa	Cnidaria	Anthozoa	Scleractinia	Merulinidae
	ingroup	*Cyphastrea salae*	GCA_964194085.1	Metazoa	Cnidaria	Anthozoa	Scleractinia	Merulinidae
**Hetmag1_Stihad2_Stimer3** *[Non-coding: No]*	outgroup	*Heteractis magnifica*	GCA_011763375.2	Metazoa	Cnidaria	Anthozoa	Actiniaria	Stichodactylidae
	ingroup	*Stichodactyla haddoni*	GCA_049996035.1	Metazoa	Cnidaria	Anthozoa	Actiniaria	Stichodactylidae
	ingroup	*Stichodactyla mertensii*	GCA_011800005.2	Metazoa	Cnidaria	Anthozoa	Actiniaria	Stichodactylidae
**Moncap1_Mongri2_Moneff3** *[Non-coding: No]*	outgroup	*Montipora capitata*	GCA_949126865.1	Metazoa	Cnidaria	Anthozoa	Scleractinia	Acroporidae
	ingroup	*Montipora grisea*	GCA_043882275.1	Metazoa	Cnidaria	Anthozoa	Scleractinia	Acroporidae
	ingroup	*Montipora efflorescens*	GCA_014634505.1	Metazoa	Cnidaria	Anthozoa	Scleractinia	Acroporidae
**Palcar1_Palmut2_Palgra3** *[Non-coding: No]*	outgroup	*Palythoa caribaeorum*	GCA_965234985.1	Metazoa	Cnidaria	Anthozoa	Zoantharia	Sphenopidae

Trio ID	Role	Organism name	Accession	Kingdom	Phylum	Class	Order	Family
	ingroup	*Palythoa mutuki*	GCA_027575235.1	Metazoa	Cnidaria	Anthozoa	Zoantharia	Sphenopidae
	ingroup	*Palythoa grandiflora*	GCA_026546935.1	Metazoa	Cnidaria	Anthozoa	Zoantharia	Sphenopidae
**Palmiz1_Palcar2_Palmut3** *[Non-coding: Yes]*	outgroup	*Palythoa mizigama*	GCA_042846405.1	Metazoa	Cnidaria	Anthozoa	Zoantharia	Sphenopidae
	ingroup	*Palythoa caribaeorum*	GCA_965234985.1	Metazoa	Cnidaria	Anthozoa	Zoantharia	Sphenopidae
	ingroup	*Palythoa mutuki*	GCA_027575235.1	Metazoa	Cnidaria	Anthozoa	Zoantharia	Sphenopidae
**Pocgra1_Pocver2_Pocdam3** *[Non-coding: No]*	outgroup	*Pocillopora grandis*	GCA_964027065.2	Metazoa	Cnidaria	Anthozoa	Scleractinia	Pocilloporidae
	ingroup	*Pocillopora verrucosa*	GCF_036669915.1	Metazoa	Cnidaria	Anthozoa	Scleractinia	Pocilloporidae
	ingroup	*Pocillopora damicornis*	GCF_003704095.1	Metazoa	Cnidaria	Anthozoa	Scleractinia	Pocilloporidae
**Porrus1_Poraus2_Porcyl3** *[Non-coding: No]*	outgroup	*Porites rus*	GCA_964035705.1	Metazoa	Cnidaria	Anthozoa	Scleractinia	Poritidae
	ingroup	*Porites australiensis*	GCA_022179025.1	Metazoa	Cnidaria	Anthozoa	Scleractinia	Poritidae
	ingroup	*Porites cylindrica*	GCA_964035525.1	Metazoa	Cnidaria	Anthozoa	Scleractinia	Poritidae
**Porrus1_Porlob2_Porlut3** *[Non-coding: No]*	outgroup	*Porites rus*	GCA_964035705.1	Metazoa	Cnidaria	Anthozoa	Scleractinia	Poritidae
	ingroup	*Porites lobata*	GCA_942486035.1	Metazoa	Cnidaria	Anthozoa	Scleractinia	Poritidae
	ingroup	*Porites lutea*	GCF_958299795.1	Metazoa	Cnidaria	Anthozoa	Scleractinia	Poritidae
**Stypis1_Pocver2_Pocdam3** *[Non-coding: Yes]*	outgroup	*Stylophora pistillata*	GCF_002571385.2	Metazoa	Cnidaria	Anthozoa	Scleractinia	Pocilloporidae
	ingroup	*Pocillopora verrucosa*	GCF_036669915.1	Metazoa	Cnidaria	Anthozoa	Scleractinia	Pocilloporidae
	ingroup	*Pocillopora damicornis*	GCF_003704095.1	Metazoa	Cnidaria	Anthozoa	Scleractinia	Pocilloporidae

Supplementary Table S7. Computational performance of the EvoSubster pipeline across 40 trios.

Wall-clock time and CPU utilization were measured with GNU /usr/bin/time -v for the complete analysis pipeline (LAST pairwise alignment, substitution counting by the Python scripts in src/count/, and visualization by the R scripts in src/visualize). Within each trio label (org1_org2_org3), org1 is the outgroup species, whereas org2 and org3 are the two ingroup species. Assembly sizes (Mbp) were obtained from the NCBI Datasets. Minimum pairwise identity is the smallest of the three pairwise percent identities reported by LAST, used here as a proxy for evolutionary divergence. Wall-clock time is given as MM:SS.ss; CPU values above 100% reflect multi-thread execution; however, utilization remained well below the 800% theoretical ceiling for 8 threads because only the LAST alignment steps are parallelized. All benchmark runs were performed on a single Linux workstation (two Intel

Xeon Silver 4216 CPUs at 2.10 GHz, 32 physical cores / 64 hardware threads, 251 GB RAM; Ubuntu 22.04, kernel 5.15) with the pipeline parallelized across 8 threads. Software versions: LAST 1648, Python 3.12.7, R 4.1.2.

trio	lineage	org1_size_Mbp	org2_size_Mbp	org3_size_Mbp	min_pairwise_identity_pct	wall_clock	cpu_pct
monCap_monGri_monEff	cnidaria	689.3	1154.1	643.3	96.13	43:21.34	316
palCar_palMut_palGra	cnidaria	812	990.5	927	95.6	36:55.25	251
acrDig_acrHya_acrSpi	cnidaria	447.5	495.2	478.5	95.2	33:30.61	234
porRus_porLob_porLut	cnidaria	569.6	646.2	541.6	94.21	33:03.04	261
acrDig_acrHya_acrMil	cnidaria	447.5	495.2	475.4	95.54	32:59.37	226
acrMil_acrNas_acrMic	cnidaria	475.4	416.4	383.9	95.74	32:10.65	191
porRus_porAus_porCyl	cnidaria	569.6	576.5	589.3	94.08	31:31.06	258
dunAxi_denCri_tubCoc	cnidaria	673.6	627.2	875.8	84.86	25:58.72	359
acrAus_acrTen_acrSpa	cnidaria	482.2	403.1	559.1	91.98	24:37.35	295
cypSal_orbFav_orbFra	cnidaria	536.3	485.5	497	87.28	21:33.49	280
pocGra_pocVer_pocDam	cnidaria	361.4	353.4	234.3	96.71	21:15.69	188
styPis_pocVer_pocDam	cnidaria	397.6	353.4	234.3	81.56	20:24.89	195
echHor_orbFav_cypSal	cnidaria	658.4	485.5	536.3	86.8	20:23.73	309
hetMag_stiHad_stiMer	cnidaria	279	447.7	294.9	94.29	18:55.67	251
casOrn_casXam_casAnd	cnidaria	458.6	326.1	406.6	82.61	17:48.72	292
palMiz_palCar_palMut	cnidaria	373.3	812	990.5	83.96	15:42.12	420
denHet_gigRos_gigMar	fungi	181.6	567.9	773.1	81.45	12:28.09	501
armBor_armGal_armAlt	fungi	71.7	86.1	73.7	88.14	6:46.37	187
funGeo_funCal_funMos	fungi	134.3	146.2	145.7	82.46	6:05.36	320
lacSan_lacDel_lacHat	fungi	86.2	96	76.7	88.82	6:01.31	280
rhiCla_rhiIrr_rhiPro	fungi	146.8	146.8	99	84.98	5:52.82	296
lacAka_lacHen_lacPse	fungi	80.3	62	99.7	89.82	5:37.43	266
lenEdo_lenLat_lenNov	fungi	45.6	45.4	35.6	93.44	5:05.62	164
pleOst_pleEry_pleTuo	fungi	34.9	53.5	43.8	89.38	4:54.67	162
bolBar_bolRet_bolNob	fungi	48.8	40.7	75.2	91.44	4:51.66	207
lacAme_lacBic_lacTri	fungi	52.2	64.9	59.1	86.59	4:40.51	186
rhiPro_rhiIrr_rhiIrr	fungi	99	146.8	161.9	84.6	4:32.54	352
podMin_podVer_podHum	fungi	39.4	41.9	36.2	83.16	4:28.26	146
agaBis_agaBit_agaSin	fungi	30.2	32.3	32.5	88.71	4:25.84	147
lecGlu_lecDis_lecPro	fungi	65.6	72	65.6	95.53	4:18.10	234
parBra_parOcc_parOcc	fungi	58.5	50.1	44.9	87.6	4:04.71	211
lecObs_lecIns_lecImi	fungi	69.7	69.6	63.6	96.59	3:58.11	234
bolRex_bolRet_bolEdu	fungi	54.2	55.8	66.5	92.39	3:34.40	262
bolSem_bolTyl_bolPse	fungi	69	54.1	56.3	85.67	3:13.77	217
strPac_strLuc_strSte	fungi	51.8	46.7	42.4	83.51	3:13.72	187
podMar_podPis_podRug	fungi	37.5	35.4	32.5	91.62	3:11.55	177
bolVar_bolEdu_bolRex	fungi	62.1	66.5	54.2	90.36	3:07.98	296
bolNan_bolTom_bolMar	fungi	43.8	45.5	47.7	96.53	3:01.52	226
rusAbi_rusGri_rusLep	fungi	53.6	50.6	41.5	80.43	2:55.27	266
inoSue_inoTig_inoFlo	fungi	44.5	42	52	84.22	2:34.44	211
